# Implementation of adverse event reporting for medical devices, India

**DOI:** 10.2471/BLT.19.232785

**Published:** 2019-11-18

**Authors:** Shatrunajay Shukla, Madhur Gupta, Sabitri Pandit, Milu Thomson, Abhimanyu Shivhare, Vivekanandan Kalaiselvan, Gyanendra Nath Singh

**Affiliations:** a Medical Device & Materiovigilance Programme of India, Indian Pharmacopoeia Commission, Ministry of Health & Family Welfare, Government of India, Sector-23, Raj Nagar, Ghaziabad-201002, Uttar Pradesh, India.; b Office of the WHO Representative to India, New Delhi, India.; c Indian Pharmacopoeia Commission, Ministry of Health & Family Welfare, Uttar Pradesh, India.

## Abstract

**Problem:**

Rapid growth in the use of medical devices has drawn attention to gaps in the systematic monitoring of medical device-associated adverse events in India.

**Approach:**

Implementation of national regulations on medical devices started in January 2018. Supported by a nationwide network of monitoring centres, the Indian Pharmacopoeia Commission coordinates adverse event reports from manufacturers, legal representatives and patients or users. The commission follows-up and reviews reports with subject expert groups and sends recommendations on necessary action to the national regulatory authority.

**Local setting:**

Before 2015, no systematic structure was in place to collate adverse events associated with medical devices. Several reports of deaths and hospitalization due to faulty hip implants, cardiac stents and poor-quality devices prompted the health ministry to launch the materiovigilance programme.

**Relevant changes:**

From July 2015 to October 2019, the commission received 1931 adverse event reports, mostly from marketing authorization holders; 1277 were serious events. Reporting increased markedly after 2017. Cardiac stents were the most reported device (926 events; 47.95%). To encourage a culture of reporting, the commission has raised awareness about the programme among stakeholders, developed user-friendly reporting tools and guidelines, and conducted training for hospital personnel on medical device adverse event reporting.

**Lessons learnt:**

Regular training to stakeholders develops a sense of responsibility towards reporting medical device adverse events and ensures quality data reporting. Reporters must be assured that reporting adverse events does not have any legal implications for them and given acknowledgement of their role in high-quality device associated adverse event reporting.

## Résumé

**Problème:**

La croissance rapide de l’utilisation des dispositifs médicaux a attiré l’attention sur les lacunes dans le suivi systématique des évènements indésirables associés aux dispositifs médicaux en Inde.

**Approche:**

Une réglementation nationale sur les dispositifs médicaux a été mise en place en janvier 2018. Soutenue par un réseau national de centres de surveillance, la Commission de la Pharmacopée indienne coordonne les signalements d’évènements indésirables effectués par les fabricants, les représentants légaux et les patients ou les utilisateurs. La Commission assure un suivi et une évaluation des signalements avec des groupes d’experts et adresse à l’autorité réglementaire nationale des recommandations sur les mesures nécessaires.

**Environnement local:**

Avant 2015, il n’existait aucune structure systématique pour compiler les évènements indésirables associés aux dispositifs médicaux. Plusieurs signalements de décès et d'hospitalisation causés par des prothèses de hanche défectueuses, des stents cardiaques et des dispositifs de mauvaise qualité ont poussé le ministère de la Santé à lancer le programme de matériovigilance.

**Changements significatifs:**

Entre juillet 2015 et octobre 2019, la Commission a reçu 1931 signalements d’évènements indésirables, principalement de la part de titulaires d’autorisation de mise sur le marché; 1277 étaient des évènements graves. Le nombre de signalements a nettement augmenté à partir de 2017. Les stents cardiaques étaient le dispositif faisant l’objet du plus grand nombre de signalements (926 évènements; 47,95%). Pour encourager une culture du signalement, la Commission a mené des actions de sensibilisation sur le programme auprès des parties prenantes, a élaboré des outils conviviaux et des directives de déclaration et a formé le personnel hospitalier au signalement des évènements indésirables associés aux dispositifs médicaux.

**Leçons tirées:**

La formation régulière des parties prenantes développe un sens des responsabilités vis-à-vis du signalement des évènements indésirables associés aux dispositifs médicaux et garantit la déclaration de données de qualité. Les déclarants doivent avoir l’assurance que le signalement d’évènements indésirables n’a aucune répercussion juridique pour eux et leur rôle dans la qualité des signalements des évènements indésirables associés aux dispositifs médicaux doit être reconnu.

## Resumen

**Situación:**

El crecimiento acelerado en el uso de dispositivos médicos ha llamado la atención sobre los vacíos en el seguimiento sistemático de los eventos adversos asociados a los dispositivos médicos en la India.

**Enfoque:**

La implementación de la normativa nacional sobre los dispositivos médicos comenzó en enero de 2018. Con el apoyo de una red nacional de centros de seguimiento, la Comisión de Farmacopea de la India coordina las notificaciones de los eventos adversos presentadas por los fabricantes, los representantes legales y los pacientes o usuarios. La Comisión realiza el seguimiento y la revisión de las notificaciones con los grupos de expertos y envía recomendaciones sobre las medidas necesarias a la autoridad reguladora nacional.

**Marco regional:**

Antes de 2015, no existía una estructura sistemática para recopilar los eventos adversos asociados con los dispositivos médicos. Varias notificaciones de muertes y hospitalizaciones por implantes de cadera defectuosos, stents cardíacos y dispositivos de mala calidad llevaron al Ministerio de Salud a poner en marcha el programa de tecnovigilancia (materiovigilance programme).

**Cambios importantes:**

Entre julio de 2015 y octubre de 2019, la Comisión recibió 1931 notificaciones de eventos adversos, la mayoría de ellas de titulares de la autorización de comercialización; 1277 fueron eventos graves. Las notificaciones aumentaron notablemente después de 2017. El dispositivo más notificado fue el stent cardíaco (926 eventos; 47,95 %). Para fomentar una cultura de notificación, la Comisión ha sensibilizado a las partes interesadas sobre el programa, ha desarrollado herramientas y directrices de fácil uso para la notificación y ha impartido formación al personal hospitalario sobre la notificación de los eventos adversos de los dispositivos médicos.

**Lecciones aprendidas:**

La formación regular a las partes interesadas desarrolla un sentido de responsabilidad hacia la notificación de eventos adversos de los dispositivos médicos y garantiza la calidad de la notificación de los datos. Quienes notifiquen eventos adversos deben saber que la notificación de los mismos no tiene implicaciones legales para ellos y que se les reconoce su participación en la notificación de eventos adversos asociados a los dispositivos de alta calidad.

## ملخص

**المشكلة:**

أدى النمو السريع في استخدام الأجهزة الطبية إلى جذب الانتباه إلى الثغرات الموجودة في المراقبة المنهجية للأحداث السلبية المرتبطة بالأجهزة الطبية في الهند.

**الأسلوب:**

بدأ تنفيذ اللوائح الوطنية الخاصة بالأجهزة الطبية في يناير/كانون ثاني 2018. وفي ظل الدعم من شبكة وطنية لمراكز المراقبة، تقوم لجنة دستور الأدوية الهندية بتنسيق تقارير الأحداث السلبية من جهات التصنيع، والممثلين القانونيين، والمرضى أو المستخدمين. تقوم اللجنة بمتابعة التقارير ومراجعتها مع مجموعات من الخبراء ذوي الصلة، وترسل توصيات حول الإجراءات اللازمة إلى السلطة التنظيمية الوطنية.

**المواقع المحلية:**

قبل عام 2015، لم يكن هناك أية بنية منهجية قيد الاستخدام لتصنيف الأحداث السلبية المرتبطة بالأجهزة الطبية. العديد من بلاغات الوفيات وتقارير العلاج بالمستشفيات، الناتجة عن أخطاء في زراعات الورك، ودعامات القلب، والأجهزة منخفضة الجودة، دفعت وزارة الصحة إلى إطلاق برنامج الاحتراز المادي.

**التغيّرات ذات الصلة:**

من يوليو/تموز 2015 إلى أكتوبر/تشرين أول 2019، استقبلت اللجنة 1931 تقريراً بخصوص أحداث سلبية، معظمها من حاملي تراخيص التسويق؛ 1277 منها كانت أحداثاً خطيرة. زادت وتيرة الإبلاغ بشكل ملحوظ بعد عام 2017. وكانت أغلب البلاغات بخصوص دعامات القلب (926 حدثاً؛ بنسبة 47.95%). لتشجيع ثقافة الإبلاغ، قامت اللجنة برفع الوعي حول البرنامج بين أصحاب المصلحة، وقامت بتطوير أدوات وإرشادات الإبلاغ سهلة الاستخدام، ونفذت تدريباً لفريق العاملين في المستشفى على الإبلاغ عن الأحداث السلبية للأجهزة الطبية.

**الدروس المستفادة:**

يؤدي التدريب الدوري لأصحاب المصلحة إلى توليد شعور بالمسؤولية تجاه الإبلاغ عن الأحداث السلبية للأجهزة الطبية، وضمان جودة الإبلاغ عن البيانات. يجب أن يضمن أصحاب البلاغات أن الإبلاغ عن الأحداث السلبية لا يترتب عليه أية آثار قانونية بالنسبة لهم، ويجب أن يحصلوا على التقدير لدورهم في الإبلاغ بجودة عالية عن الأحداث السلبية المرتبطة بالجهاز.

## 摘要

**问题:**

医疗器械使用的快速增长引起了人们对印度医疗器械相关不良事件系统监测方面差距的关注。

**方法:**

2018 年 1 月开始实施印度《医疗器械法规 2018》。在全国监测中心网络的支持下，印度药典委员会协调来自制造商、法定代理人和患者或用户的不良事件报告。印度药典委员会与项目专家组一起跟踪和审查报告，并向国家监管机构提出必要的行动建议。

**当地状况:**

2015 年之前，缺乏系统性结构来整理与医疗器械相关的不良事件。几份有关髋关节植入失误、心脏支架和劣质设备导致死亡和住院的报告促使卫生部启动了物资警戒计划。

**相关变化:**

从 2015 年 7 月至 2019 年 10 月，委员会收到了 1931 份不良事件报告，大部分报告来自销售许可持有人；其中，1277 份为严重事件。2017 年后，报告数量显著增加。心脏支架是被报告次数最多的器械（926 起事件；47.95%）。为了鼓励形成报告文化，委员会提高了利益相关者对该方案的认识，开发方便用户的报告工具和准则，并就医疗器械不良事件报告对医院工作人员进行培训。

**经验教训:**

对利益相关者的定期培训提高了报告医疗器械不良事件的责任感，并确保了高质量的数据报告。必须向报告者保证，报告不良事件对他们没有任何法律影响，并认可他们在高质量器械相关不良事件报告方面的积极作用。****

## Резюме

**Проблема:**

Стремительный рост использования медицинских устройств в Индии привлек внимание к пробелам в систематическом мониторинге нежелательных явлений, связанных с использованием медицинских устройств.

**Подход:**

Внедрение государственных норм в отношении применения медицинских устройств началось в январе 2018 года. Индийская фармакопейная комиссия, поддерживаемая общегосударственной сетью центров мониторинга, координирует сбор информации о нежелательных явлениях, связанных с использованием медицинских устройств, от производителей, юридических представителей и пациентов или пользователей. Комиссия отслеживает и рассматривает отчеты с участием групп экспертов в данной области и направляет рекомендации относительно необходимых действий в национальный регулирующий орган.

**Местные условия:**

Структурированная система для сопоставления нежелательных явлений, связанных с использованием медицинских устройств, отсутствовала вплоть до 2015 года. Несколько случаев смерти и госпитализации по причине неисправности имплантатов бедра, сердечных стентов и некачественных устройств побудили Министерство здравоохранения внедрить программу контроля за инцидентами, связанными с использованием медицинских изделий.

**Осуществленные перемены:**

С июля 2015 года по октябрь 2019 года комиссия получила 1931 отчет о нежелательных явлениях, в основном от держателей регистрационных удостоверений; 1277 из них были серьезными нежелательными явлениями. Количество отчетов заметно возросло после 2017 года. Наибольшее количество отчетов было зарегистрировано для сердечных стентов (926 случаев; 47,95%). В целях стимулирования системы информирования о нежелательных явлениях комиссия активно привлекает внимание заинтересованных сторон к программе, разрабатывает удобные в использовании инструменты и руководящие принципы информирования, а также проводит обучение персонала больниц методам отчетности о нежелательных явлениях, связанных с использованием медицинских устройств.

**Выводы:**

Регулярное обучение заинтересованных сторон прививает чувство ответственности в отношении информирования о нежелательных явлениях, связанных с использованием медицинских устройств, и обеспечивает качественную отчетность. Сообщающие о нежелательных явлениях лица должны быть уверены в том, что информирование о таких явлениях не влечет за собой никаких правовых последствий, и получать благодарность за их вклад в поддержание системы качественного информирования о нежелательных явлениях, связанных с применением устройств.****

## Introduction

Rapid growth in the use of medical devices in health-care settings has been enabled by technological advancements, such as drug–device combination products, automation and wireless technology, and advanced clinical application of devices.^1^ The estimation of the global market for medical devices increased from 260 billion United States dollars (US$) in 2006 to over US$ 380 billion in 2016.^2^ The quality of devices, however, can vary and even the best-designed products can fail in clinical practice. Post-market surveillance is therefore essential to ensure the quality and evaluate the safety and performance of medical devices. Despite the importance placed on surveillance of drug safety, the need for better monitoring of medical device-associated adverse events receives less attention.

A well-structured vigilance system is the backbone of a robust regulatory framework to ensure the quality and promote the safe use of medical devices. The regulation of medical devices, however, is a complex and evolving area that is often complicated by legal technicalities. For example, legal terminologies are sometimes non-uniform even within the same regulatory system. Regulations may differ from one country to another. Here we report our experiences with the design of a system to monitor the safety of medical devices in India.

## Local setting

Earlier, medical devices were regulated under the Indian Drugs and Cosmetics Act and no systematic structure was in place to collate adverse events associated with medical devices. Several reports^3^ of deaths and hospitalization due to faulty hip implants, cardiac stents and poor-quality devices, drew attention to the need for a parallel system for surveillance of medical devices. In July 2015, the Indian health ministry approved the establishment of the materiovigilance programme, with the Indian Pharmacopoeia Commission as the national coordinating centre. In 2017, the government of India issued the Medical Devices Rules 2017 for regulating medical devices used throughout the country.^4^ The rules came into effect on 1 January 2018. 

## Approach

The materiovigilance programme aims to enable data collection and evaluation in a systematic manner so that regulatory decisions and recommendations on the safe use of medical devices in India can be evidence-based. The programme also aims to create awareness among stakeholders about the importance of medical device adverse event reporting and to monitor the benefit–risk profile of medical devices.

The Central Drugs Standard Control Organization, under the directorate general of health services of the health ministry is the national regulatory authority responsible for approval of the manufacturing, import, labelling, sale and distribution of medical devices, including in-vitro diagnostics, and the conduct of clinical trials.^5^ The organization drew up an initial priority list of medical devices to be regulated under the Medical Devices Rules 2017 (Box 1) and will add other devices to the list over time.

Box 1 **Medical devices regulated under India’s national regulations**

The following medical devices and in-vitro diagnostics are currently regulated in India: cardiac stents, drug-eluting stents, catheters, heart valves, orthopaedic implants, intraocular lenses, intravenous cannulae, bone cements, ablation devices, internal prosthetic replacements, intrauterine contraceptive devices, condoms, tubal rings, umbilical tapes, blood sera, scalp vein sets, ligatures, sutures, staplers, surgical dressings, disposables syringes, hypodermic needles, perfusion sets and in-vitro diagnostic tests for human immunodeficiency virus, hepatitis B surface antigen and hepatitis C virus.

After the launch of the programme, the Indian Pharmacopoeia Commission started developing user-friendly reporting tools, technical documents and manuals, and conducting district- and zone-level training for stakeholders on the importance of reporting medical device adverse events, and use of the tools available. The reporting tools include forms for event reporting and recall or field safety corrective action and, more recently, an online reporting form that can be filled electronically. A toll-free telephone number was also made available for patients and users to report events.^6^ Patient follow-up or any further information may also be shared through these tools. The commission started building a network of monitoring centres across the country and recruiting subject expert groups.

At regional level, the materiovigilance framework is organized around health-care facilities, manufacturers and their legal representatives, and any individual aware of an incident incurring risk for themselves or others. The commission receives reports of medical device adverse events from throughout the country, including from notified medical device adverse event monitoring centres, adverse drugs reaction monitoring centres and marketing authorization holders. The commission has two partner organizations; the Sree Chitra Tirunal Institute for Medical Sciences and Technology provides the research and development facility to test medical devices and the National Health System Resource Centre assists with developing standard operating procedures and guidance manuals and in identifying monitoring centres.

At present, the materiovigilance programme has 26 dedicated medical device adverse event monitoring centres across India, which report events spontaneously on a voluntary basis. In addition, more than 270 adverse drugs reaction monitoring centres established under the pharmacovigilance programme have also been requested to report adverse events on medical devices. Once the commission enrol a medical institute or hospital as a monitoring centre, a research associate at the monitoring centre starts collating and sending medical device adverse events to the commission. Research associates liaise between the commission and patient or user, record and validate any reported incident, recommend precautionary measures as appropriate, report incidents to the commission and also inform the medical device manufacturer. Any suspected serious adverse events and action taken, including recall of devices, must be reported to the commission and the Central Drugs Standard Control Organization within 15 calendar days of the event becoming apparent. The commission also receives voluntary reports of non-serious incidents from any person. 

**Fig. 1:**
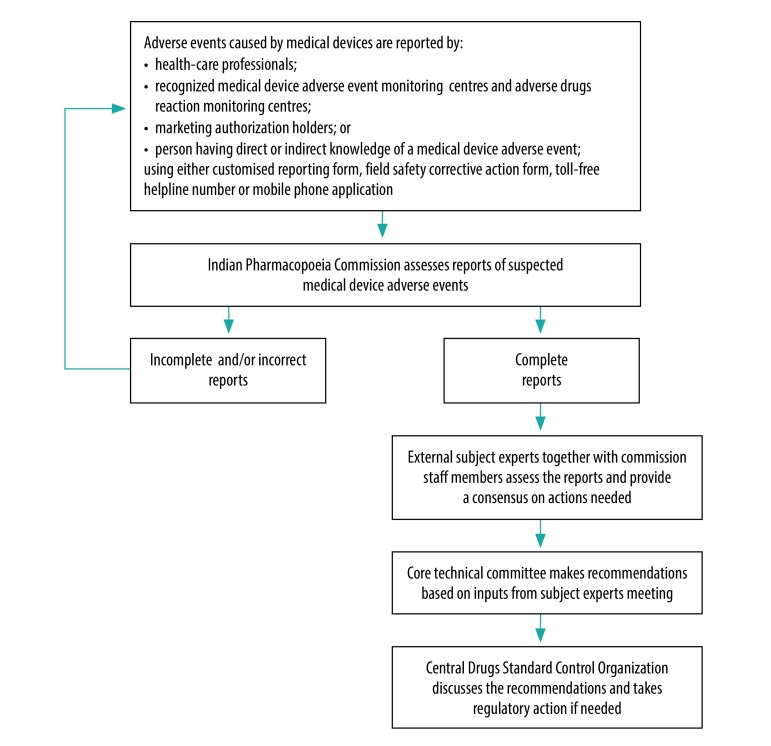
Flowchart of India’s materiovigilance programme

Note: Indian Pharmacopoeia Commission is the national coordination centre for materiovigilance programme. Central Drugs Standard Control Organization is the national regulatory authority for the manufacturing, import, labelling, sale and distribution of medical devices.

At the commission, each report received is segregated into initial, follow-up or final and allotted a unique reference number. Regarding initial and follow-up reports, the commission further seeks information from the reporter or patient until the conclusion is reached. Trained professional staff at the commission then assess these reports for quality and completeness of data and, if found valid, they are further evaluated by a group of external subject experts and sent to the core technical committee to prepare any necessary recommendations. The recommendations of the core technical committee are forwarded to the Central Drugs Standard Control Organization for further discussion and regulatory action, if any. If the data are incomplete or invalid, reports are relayed back to the relevant monitoring centre or reporter with the query or necessary comments, so that the report can be corrected or completed and returned to the commission for evaluation (Fig. 1).

The commission also receives recall and field safety notices from other regulatory agencies worldwide, such as the United States Food and Drug Administration, the Therapeutic Goods Administration of Australia, Health Canada and the Medicines and Healthcare Products Regulatory Agency, United Kingdom of Great Britain and Northern Ireland. These notices are circulated to all monitoring centres to check whether the same or similar devices are available in their local health-care organizations.

Medical device adverse event monitoring centres are now obliged to organize advance-level training for hospital personnel in their respective region and continuing medical education training in materiovigilance to increase awareness about the programme. Training programmes for the professionals involved in data collection, processing and analysis are organized periodically to develop competency for assessing the cause and performing root cause analysis for adverse events. The trainings are designed by the commission with the help of partner organizations and delivered by staff from the commission, the Central Drugs Standard Control Organization, partner organizations and industry representatives in one-day (basic level) or two-day (advanced level) courses, depending on stakeholder’s needs.

## Relevant changes

From the start of the materiovigilance programme in July 2015 up to October 2019, the Indian Pharmacopoeia Commission has received and analysed more than 1931 medical device adverse events, 1277 (66.1%) of which were serious. Reporting of device-associated adverse events in India increased markedly after 2017, when the Medical Devices Rules came into effect and after the development of various user-friendly reporting procedures (Box 2). Reported events mostly concerned medium to high-risk category medical devices that were known and procedural errors. Adverse events associated with cardiac stents were the most commonly reported (926 events; 47.95%), followed by intrauterine contraceptive devices and orthopaedic implants. Most of the events (1439; 74.5%) were received from marketing authorization holders.

Box 2 **Medical device adverse events reported to the Indian Pharmacopoeia Commission**

A total of 1931 adverse events were reported from July 2015 to October 2019:

- 40 events in 2015; 53 in 2016; 254 in 2017; 687 in 2018; and 897 in 2019 (till October 2019).
- 1277 events were classified as serious and 654 as non-serious.^a^
- 926 events were associated with cardiac stents; 226 with intrauterine contraceptive devices; 179 with orthopaedic implants; 75 with intravenous cannulae; 76 with catheters; and 449 with other types of device.
- 1439 events were reported by marketing authorization holders; 419 by medical device adverse event monitoring centres; 70 by adverse drug reaction monitoring centres and 3 by consumers.

^a^ Criteria for seriousness of events are defined in the Indian Medical Devices Rules 2017.

An example of how the data are used to address safety concerns is expulsion of intrauterine contraceptive devices and genital haemorrhage, which is found to be related to devices. During analysis of adverse event reports from different locations, the commission observed they were from the same faulty batch of devices supplied by the manufacturer. The findings were communicated to the Central Drugs Standard Control Organization to act and to monitoring centres for further surveillance of similar cases. 

## Lessons learnt

Setting up a medical device vigilance system in a low-middle income country of more than 1.36 billion people involves several challenges. Over the past 4 years, the Indian Pharmacopoeia Commission has gained considerable knowledge about how to develop the necessary tools and reporting culture for medical device surveillance. In the early implementation phase, stakeholders were largely unaware of the materiovigilance programme and its requirements and procedures. Therefore, the commission needed to start providing formal training under the programme to stakeholders through its network of partner organizations (Box 3). Since there are many different stakeholders in the programme with a need for and interest in materiovigilance, training had to be designed at different levels, with different content and for different time periods. At the regional level, regular training to health-care professionals was needed to foster a sense of responsibility and generate awareness on what, how and where to report medical device adverse events. To counter any reluctance to report adverse events, health-care professionals need to be assured that submitting a report does not have any legal implications for them and be given acknowledgement of their role by the commission. Awareness-raising through dissemination of technical documents, manuals and newsletters also helped the sustainability of the system. Set up in 2018, the toll-free helpline number for guidance of patients and users when submitting reports, helped to improve issues with low medical device adverse events reporting.

Box 3 **Summary of main lessons learnt**

• Identification of monitoring centres and proactive on-site assessment and capacity-building through regular basic and advanced level training expands the reach of the programme and ensures quality data reporting.

• Regular training to health-care professionals about the materiovigilance programme develops a sense of responsibility and raises awareness on what, how and where to report medical device adverse events.

• Reporters of medical device adverse events must be assured that reporting does not have any legal implications for them and be given acknowledgement of their role in high-quality reporting by the regulatory authority.

## Next steps

The online medical device adverse events reporting form^7^ is currently in English language only. To enhance participation from regional patients and users, the commission is working towards providing the form in other languages commonly used in India (Hindi, Punjabi, Bengali, Tamil, Telugu, Malayalam, Gujarati and Marathi). A mobile phone application for reporting device associated events is also under development that will enhance access to the materiovigilance programme. 

Unlike for drugs, there is no database for medical device adverse events in India. Developing a national database for analysing and management of adverse event reports will facilitate the coding of medical device adverse event terminology. This database should include the statistical tools to support calculation of proportional reporting ratios and the information component for signal detection. Engaging consumer societies and patient-based organizations for reporting adverse events may also be considered. At the national level, provisions related to post-market surveillance of medical devices are a prerequisite. Reporting events to the regulatory authority should be mandatory for marketing authorization holders, especially concerning serious adverse events and recalls, while being voluntary for patients and users. Introduction of education on vigilance of medical products at undergraduate and postgraduate levels in academic institutions for health-care professionals is recommended.

To further enhance the reporting culture in India, the commission is identifying new monitoring centres and strengthening its staff in public and private hospitals and research centres across the country. Since the data provided by monitoring centres will contribute to regulatory decisions, the commission is planning to implement inspections or audits of monitoring centres to ensure the quality of reported cases. The commission, along with the Central Drugs Standard Control Organization, is continuing to make it mandatory to submit periodic safety usage reports on medical devices to the commission and the Central Drugs Standard Control Organization to monitor the quality and safety aspects of medical devices used in India.

## References

[1] Fouretier A, Bertram D. New regulations on medical devices in Europe: what to expect? Expert Rev Med Devices. 2014 7;11(4):351–9. 10.1586/17434440.2014.916209 24811927

[2] Ted Fuhr EM, Silverman S, Telpis V. Capturing the value of good quality in medical devices [internet]. New York: McKinsey & Company; 2019. Available from: https://www.mckinsey.com/industries/pharmaceuticals-and-medical-products/our-insights/capturing-the-value-of-good-quality-in-medical-devices [cited 2019 Nov 6].

[3] Woman who sued Johnson & Johnson for faulty implant dead [internet]. The Times of India. 2014 May 12. Available from: https://timesofindia.indiatimes.com/city/mumbai/Woman-who-sued-Johnson-Johnson-for-faulty-implant-dead/articleshow/34986373.cms [cited 2019 Nov 6].

[4] Bhave A. Indian regulatory update: January–December 2017. Perspect Clin Res. 2018 Jan-Mar;9(1):48–50. 10.4103/picr.PICR_176_17 29430419 PMC5799954

[5] Gupta SK. Medical device regulations: a current perspective. J Young Pharm. 2016;8(1):6–11. 10.5530/jyp.2016.1.3

[6] Kalaiselvan V, Mishra P, Singh GN. Helpline facility to assist reporting of adverse drug reactions in India. WHO South-East Asia J Public Health. 2014 Apr-Jun;3(2):194. 10.4103/2224-3151.206737 28607307

[7] Medical devices adverse event reporting tools [internet]. Ghaziabad: Indian Pharmacopoeia Commission; 2019. Available from: https://ipc.gov.in/mandates/pvpi/materiovigilance-programme-of-india-mvpi.html?id=597:medical-devices-adverse-event-reporting-tools&catid=2 [cited 2019 Oct 31].

